# Supplementary material for: NKX2-5 mutations causative for congenital heart disease retain functionality and are directed to hundreds of targets
Source: eLife. 2015 Jul 6;4:e06942. doi: 10.7554/eLife.06942 (PMC4548209; doi:10.7554/eLife.06942)
Supplement: Supplementary file 2. — Supplementary tables. List of antibodies (A) and primers used for qPCR and ChIP (B). (C) Peak calling from three independent Dam experiments. DOI: http://dx.doi.org/10.7554/eLife.06942.030 [file elife06942s002.docx]

### Supplementary File 2

### A: List of antibodies used in this study

| **Antigen** | **Supplier** | **Catalogue #** |
| --- | --- | --- |
| **IgG control (ChIP)** | Santa Cruz Biotechnology | Sc-2025 |
| **NKX2-5 (ChIP)** | Santa Cruz Biotechnology | Sc-365207 |
| **NKX2-5 (western blotting)** | Santa Cruz Biotechnology | Sc-8697 |
| **tElk1** | Cell Signaling Technology | 9182 |
| **pElk1** | Cell Signaling Technology | 9181 |
| **V5-tag** | Invitrogen | R960-25 |
| **HA-tag** | Cell Signaling Technology | 3724 |

### B: List of primers used in this study for qPCR and ChIP (*)

| **Gene** | **Forward primer** | **Reverse Primer** |
| --- | --- | --- |
| *Abcb8* | TCCGGGGCCSGGTCSTSGGTTTC | TCGTGGGCSTTGGCTTCTCGTG |
| *Actb* | ACCAACTGGGACGATATGGAGAAGA | TACGACCAGAGGCATACAGGGACAA |
| *Actc1* | AGAGTATGATGAGGCAGGCC | ATGACTGATGAGAGATGGGG |
| *Adrb1* | CTCGGCCTTCAACCCCATCATCTA | CGCCCCGGCTCGTCCAG |
| *ANF/Nppa* | TTCTTCCTCGTCTTGGCCTT | GCCTACGAAGATCCAGCTGCTT |
| *Ankrd54* | CCCTGCGCAGCGGATGATAAAG | GGGCCCCTCCTCGAAGCAGTG |
| *Atoh8* | CACGGCTGGCTGACCTGGACTA | GCGGCCCTTGTGCTGGTTCTAA |
| *Bcl2* | ATGCCAACGGGGAAACACCAGAA | CCCACGGCCCCGAAAGAAA |
| *Bmpr1a* | CGAGCGCCGGAGGATGAGTT | GTCCTGTACACGGCCCTTTGAATG |
| *Btg1* | TCTGCCGCTTTCTGGTTCTCATCT | TGCCCCTCCCCCAATCCAGTTA |
| *CCnj* | CCCCCTGCCCGCCATCTC | AGCCGTCGTGAAGGTCTGTCTCG |
| *Crim1* | AGCCGGGCCACCTGTCCTGT | CGGGGGCGTGGCAAATAGAAG |
| *Cyr61* | AGAGTGCCGCCTGGTGAAAGAGAC | GTTGGGATGCGGGCAGTTGTAGTT |
| *Deptor* | GTCGGAGGCGGAGGCTGATG | AGTGTGGGGCTGCTGCTGAAGTAG |
| *Dnajc8* | CCTCCCCGCTGCTCCTCCTG | GAAACCAAGCCCCTCCTCTCAATG |
| *Dock4* | GGACTGTCATGCGCCTGGTTGA | ATTCCGGAGATCTGGCTGGGGTAT |
| *Dsp* | GCCACCCCAAGTTATCTAAACA | GTTTGTCCCCTGTAGACTCCTG |
| *Etfb* | GATACCAGGGGCACAGGCAGAAAG | TGTTGGGCAGGGTGGCATAGC |
| *Fbxo5* | AAGAAAATGGGCCTGGAGCACCTA | ACGCCCCCTTATTGTTTTCCAGTA |
| *Gata4* | GATATGGGTGTTCCGGGTTGTTCA | CATGGCCAGGCTGCGATTTG |
| *Gemin8* | CATCCCCGTGGCTCTTCCCTCAT | ACCGTGCTTCATTCCCTCTTGTGG |
| *Hand2* | CCCGCCGACACCAAACTCTC | CCCCCGGCTCACTGCTCTC |
| *Hint1* | CATTTCCCCTCAAGCACCAACACA | GTCCCCCGTCTGCACCTTCATTC |
| *Hist1h2bg* | GGAGGGCACCAAGGCTGTCACTAA | ATCTGGCAGCGCGTTGGTTACACT |
| *Hist1h2bh* | CCCGCCAAGTCCGCTCCTG | CGCTTCTTGCCGTCCTTCTTCTG |
| *Hist1h3g* | ACCGGCGGCGTGAAGAAGC | TGGATGGCGCACAGGTTGGT |
| *Hist3h2bb-ps* | GGGCGTCCGCGTTTCCGTAGTA | CCGTTTGCGTTTCTTGCCATCCTT |
| *Hnrnpc* | TGGCGAGGATGGCAGAATGAT | GTAGAGGGGACGGAGAAGGGTGTT |
| *Ict1* | GGCCAGCCAGGTACCCAAAGAGC | GCAGGGCGGTTAATCGAGAAAAGT |
| *Id2* | GCCGCTGACCACCCTGAACAC | AGAACGACACCTGGGCAAGACGA |
| *Id3* | GCTGCCTGTCGGAACGTAG | TCAGTGGCAAAAGCTCCTCT |
| *Ift46* | CTGCCTCCCGTTGCCATTCC | GCCCCAACCCGACCCTACTCAC |
| *Kctd10* | GGCCGCCCTACAACAGAACAAAGA | GGCCGTAAAATGACCAACAGCAGA |
| *Myl1* | CTGCGGGTCAAGCGAGTCACA | GCGGGCTTCTTCACGTCTTTCTTT |
| *Ngdn* | GGACGCTCGACACCCTCACG | ATCACCAGCGCCTCCGAAAACC |
| *Ninl* | AGCTGCCGGCACTCCTTCACATT | GCTCCGACGGCCATACCACTTAGA |
| *Nkx2-5 3’UTR* | CTCCGATCCATCCCACTTTA | AGTGTGGAATCCGTCGAAAG |
| *Nkx2-5 ORF* | CCCAAGTGCTCTCCTGCTTTC | TCCAGCTCCACTGCCTTCTG |
| *P2Ry6* | CACTGGGGCCATGGTTGAAGC | GGGGGTGGGGGTGGAGAAGA |
| *PawR* | ATGCGCCGGCTAGTTTCTCCTCAA | CCGCCCGTGCAGCTTCTACCTT |
| *Ryr2* | CGAGCGCTGCCAAAGACCTACA | TCTTTGCCCATCCTCACACTCAGC |
| *Sema5a* | ACGACCTGTGGCATTGGCTTCC | CTCCGGGCATGCATTGGTGTTAC |
| *Smarcd3* | CCGGCTGCTGGGGTTACACA | TTAGGCGGGGGCAGTCAAAAAT |
| *Snx13* | AGGATCCTGCCCCGTCTCGTT | CACACTCTGGCACTCTGGCACTG |
| *Snx14* | GGCCCTGAGAAGTCGAAGAGATGA | CAGGAATGGAACCAAAGGGGAAGC |
| *Sparc* | GCTCGCCTCTAAACCCCTCCACA | CCACCACGGTTTCCTCCTCCACTA |
| *SSr3* | CCCGGAAGGAGAAAGACGAAAGA | CACCCCGAGATACAAAGGCAGAGG |
| *Suz12* | GCGGCTTCGGGGGTTCG | GACGTGCTCCATTTTCGGCTTCTT |
| *Tanc2* | GCCCAGCATTACCACGGAGTC | CGCCTGCGCATTTCTGGAGTTC |
| *Tbp* | TGTACCGCAGCTTCAAAATATTGTAT | AAATCAACGCAGTTGTCCGTG |
| *Tbx20* | ATCGCCGCGCTTATGTCCAG | CCCCGCCGCCAAACTCC |
| *Tbx3* | GACCGGCATCCCTTTCTCATCC | CCTTACCGGCCACCATCCAC |
| *Tbx5* | CTACCCCGCGCCCACTCTCAT | TGCGGTCGGGGTCCAACACT |
| *Thbs4* | ATTTGGCTCCGGGTTTCAGATGTG | GGGTTCCGGCAGCTCCTTTCA |
| *Tnni1* | CATGCCGGAAGTTGAGAGGAAATC | CCGGCGCAGGGGAGGAC |
| *Tom1l1* | AGGTCGGGAGATGCAGGAGAGGAT | ACTGGCACGATGGGGCTTAGGTC |
| *Trim17* | GTCCTCCCCGCGGCTATCAAGA | CTCGGGCGGTGGGCTCAAGTA |
| *Vcpip1* | ACAGCGGCACGAGCAGCAACA | GAAGGCCCGGTCACCAAGTAACG |
| *Wnt11* | CTGGCCCGTTTCCCTGTATGTGAT | CTTCCCCAACGTCCAGGCTTAGA |
| *Zfpm2* | TTCTTAACCCAGCCGCTCATTCAT | AGGGACCGCTGGGATTTTTCACAT |
| *ßMaj** | ACCGAAGCCTGATTCCGTAGAGC | GATGTCTGTTTCTGGGGTTGTGAGTC |
| *Btg1** | CAGTGTTTGCCACAGTGTCTTT | AAACAATGCTCAGGACGGTAGT |
| *Dsp** | GCCACCCCAAGTTATCTAAACA | GTTTGTCCCCTGTAGACTCCTG |
| *Gata4** | GCAAGTGTTGGTGAAACCATAA | TGGATCTTCAGTTCCACAAGAA |
| *Id3** | CTCTTATCCTCTTTCCCCTGGT | ACTCACTGGCGTCAGGAATTAT |
| *Mtr** | CAGGATTTACCAGAGGACAAGG | AGGCTGACAGATGCCTTTAATC |
| *Nkx2-5** | CCTTATACCGTGGAGCAGAGCC | TGTTCCCCTATTAGCATCAGAGGTT |
| *Nppa** | TAGATCTGCCCTCTTGAAAAGC | TGACCTCTATCCCATTTCCATC |
| *Ryr2** | CTTTCCCTGCCTACCACACTAT | GTTCTTTAAACGCTGGCTTGTT |
| *Tbx20** | TTTCTAAGGCCAAAGCAAAGAC | CTTTAGTGCCTGCTCTCCAGAT |
| *Tnni1** | CCTGACCAACATCACACATCTT | TCTGAGAGAGCAGAACTGTGGA |
| *Vim** | CAAGACTCAGGCACGTATCAAC | GGTTTGAGAACCCAAGCATAAG |

### C: Peak calling from three independent DamID experiments

| **Dam-fusion protein** | **# Peaks** | **Max. FDR (c=3.5)** | **# Overlapping unique peaks** | **p-value of overlap^1^** |
| --- | --- | --- | --- | --- |
| Dam-Nkx2-5^2^ | 1534 | 0.08 | N/A | |
| Dam-Nkx2-5Y191C^2^ | 1161 | 0.16 | N/A | |
| Dam-Nkx2-5ΔHD^2^ | 794 | 0.10 | N/A | |
| Dam-Elk1^3^ | 1804 | 0.12 | 1219 | 0.001 |
| Elk1-Dam^3^ | 2103 | 0.04 |  |  |
| Dam_Elk4^3^ | 1401 | 0.30 | 875 | 0.001 |
| Elk4_Dam^3^ | 1366 | 0.18 |  |  |
| Dam_SRF^3^ | 1635 | 0.06 | 1216 | 0.001 |
| SRF_Dam^3^ | 1822 | 0.01 |  |  |
| Dam-Nkx2-5^4^ | 1573 | 0.04 | N/A | |
| Dam-Nkx2-5YRD^(Y-A)4,^ ^ | 1030 | 0.04 | N/A | |

^1^determined using the Cooccur hybrid method implemented in R (100 permutations) ([Huen and Russell, 2010](#_ENREF_36)).

^2^Experiment #1 (triplicates).

^3^Experiment #2 (triplicates).

^4^Experiment #3 (quadruplicates)

^One sample was removed after QA
